# Social insights on the implementation of One Health in zoonosis prevention and control: a scoping review

**DOI:** 10.1186/s40249-022-00976-y

**Published:** 2022-05-03

**Authors:** Junyi He, Zhaoyu Guo, Pin Yang, Chunli Cao, Jing Xu, Xiaonong Zhou, Shizhu Li

**Affiliations:** 1 National Institute of Parasitic Diseases, Chinese Center for Disease Control and Prevention (Chinese Center for Tropical Diseases Research), NHC Key Laboratory of Parasite and Vector Biology, WHO Collaborating Centre for Tropical Diseases, National Center for International Research on Tropical Diseases, Shanghai, 200025 China; 2 School of Global Health, Chinese Center for Tropical Diseases Research, Shanghai Jiao Tong University School of Medicine, Shanghai, 200025 China

**Keywords:** One Health, Zoonotic diseases, Social impacts, Social perspectives

## Abstract

**Background:**

The One Health (OH) concept has been promoted widely around the globe. OH framework is expected to be applied as an integrated approach to support addressing zoonotic diseases as a significant global health issue and to improve the efficiency and effectiveness of zoonosis prevention and control. This review is intended to overview the social impact of the implementation of OH on zoonosis prevention and control.

**Methods:**

A scoping review of studies in the past 10 years was performed to overview the integration feature of OH in zoonosis prevention and control and the social impacts of OH. PubMed and Web of Science were searched for studies published in English between January 2011 and June 2021. The included studies were selected based on predefined criteria.

**Results:**

Thirty-two studies were included in this review, and most of them adopted qualitative and semi-qualitative methods. More than 50% of the studies focused on zoonosis prevention and control. Most studies were conducted in low- and middle-income countries in Africa and Asia. Applying OH approach in diseases control integrates policymakers, stakeholders, and academics from various backgrounds. The impact of OH on economic is estimated that it may alleviate the burden of diseases and poverty in the long term, even though more financial support might be needed at the initial stage of OH implementation. OH implementation considers social and ecological factors related to zoonosis transmission and provides comprehensive strategies to assess and address related risks in different communities according to regions and customs.

**Conclusions:**

Based on reviewed literature, although there seems to be a lack of guidelines for assessing and visualizing the outcomes of OH implementation, which may limit the large-scale adoption of it, evidence on the contributions of implementing OH concepts on zoonosis prevention and control indicates long-term benefits to society, including a better integration of politics, stakeholders and academics to improve their cooperation, a potential to address economic issues caused by zoonosis, and a comprehensive consideration on social determinants of health during zoonosis prevention and control.

## Background

With rapid development and complex patterns of change in various aspects worldwide, global health these days seems to be confronted by a series of “wicked problems”. “Wicked problems” are difficult or impossible to tackle due to multiple interdependent factors involving which are incomplete, constantly changing and difficult to define [1]. For global health issues we face nowadays, problems such as climate change, famine, sexually transmitted disease, zoonotic diseases and etc. are all examples of “wicked problems”. Because they are complex, multifaceted and strongly affected by social, economic and political factors, the complexity in addressing these problems makes them one of the ongoing wicked problems in global health [2]. Among these problems, zoonotic diseases are becoming increasingly prevalent. Zoonotic diseases are caused by various harmful germs such as viruses, bacteria, parasites and etc. [3]. They can be transmitted to humans either directly through non-human animals or indirectly through intermediate hosts such as mosquitoes and ticks, both leading to an emerge or re-emerge of infectious disease such as Zika, West Nile, Avian influenza, and etc. [3]. Moreover, multiple environmental factors affect host–pathogen interactions and disease dynamics [4], demonstrating the need for a holistic and collaborative approach with consideration of animal health and environmental quality when tackling zoonosis issues and achieving optimal human health.

Therefore, it seems to be appropriate to adopt the concept of One Health (OH) which emphasized the interactions between animal, human and their environments in zoonosis prevention and control [4]. OH recognizes the interconnectivity of all life-systems on earth and the connection between the health of humans, animals and the environment [5], which make OH an integrated approach to human and animal health with their respective social and environmental contexts essential [5, 6]. Also, in achieving the Sustainable Development Goals 2030 [7], where the links between human, health, climate, and ecosystems are emphasized, the OH approach has emerged and gradually played a central role by generating positive effects, such as promoting human health while closely considering the interaction on the human-animal-environment interface [8]. Multiple international organizations, including World Health Organization (WHO), World Organization for Animal Health (OIE) and Food and Agricultural Organization of the United Nations (FAO), negotiated and discussed the importance of OH in the configuration of their mandates and actions as a collaborative, multi-sectoral, and multidisciplinary approach that aims to work at local, regional, national, and global levels [9, 10]. Although OH is not a new concept to deal with global health issues, using OH as an integrated approach or a policy concept has become increasingly important and has been widely supported by various academics [9].

In parallel with the worldwide promotion of OH, an emerging body of social science studies have raised questions about how successful OH approach has been in the context of addressing global health issues for the society [11–13]. A social scientist Michalon who is a OH enthusiast and advocate referred to OH as an ‘epistemic watchword’[9], for the reasons that OH calls upon various actors to engage to produce new perspectives and knowledge and to finally achieve the core aim of global health. Some researchers agree that OH can lead to better interventions by synthesizing knowledge from various disciplines, while others argue that OH should pay more attention to the social and economic drivers of disease to achieve sustainable goals of health ultimately [11]. Although OH is advocated as an integrated approach, methodology, approach, movement, strategy, or paradigm, critical views and enquiries of OH are growing [12, 14].

‘Integration’ is a core theme in OH approach with respect to actors belonging to the domains of animal, human and environmental health [15]. OH involves the cooperation of leaders, stakeholders, managers, scientists, specialists, clinicians, epidemiologists, and statisticians [16]. Nonetheless, collaboration among these professionals can be challenging, because of factors such as professional competition, conflicting priorities, and institutional inertia, limiting the implementation of OH approach [11, 12]. OH also involves public engagement, policy-making, politics and legislation through the perspective of social sciences [16]. In addition, the efficacy of the global OH agenda to meet national and local needs should be explored. Thus, this review is intended to overview the integration feature of OH framework and impacts of OH implementation on economics and social determinants of health.

## Method

The study employed a scoping review method which can help provide a descriptive overview of the collected information from literature [17]. For this review, aiming at OH implementation and its social-related impacts on zoonosis prevention and control, scoping review may identify key contents and gaps in related research [18, 19]. Specifically, this review is intended to overview studies published in the past 10 years to investigate the integration reflected from zoonosis prevention and control through implementing OH approach and social determinants of health that were considered during OH implementation.

The review is intended to answer the following questions:

- What kind of integration was reflected from OH implementation in zoonosis prevention and control?
- What impacts on social determinants of health that can be seen from OH implementation on zoonosis prevention and control?

### Search strategy

In order to select studies that best fit the research aims, although OH and zoonosis may consist of various approaches and numerous diseases, this review is intended to include studies that clearly mentioned and interpreted OH approach and zoonotic diseases. PubMed, and Web of Science were searched for studies published in English from January 2011 to June 2021, and key terms including ‘One Health’, ‘Integrat*’, ‘Social Science’, ‘Social determinant’, ‘Social dimension’, ‘Zoonotic diseases’ and ‘Zoonosis’ were used in different combinations to identify potential studies.

### Inclusion criteria

To select studies with ‘best evidence’, literature included in the review should firstly be germane to research topic; secondly utilise adequate methodology with minimal bias; and thirdly have balanced external and internal validity [20]. These principles were applied to set the inclusion and exclusion criteria to select literature with the most relevant evidence. Inclusion criteria for literature was defined as follows:

1. Studies published between January 2011 and June 2021.
2. ‘One Health’ and ‘zoonotic diseases’ were used as the main concepts or terms in the study with definition or interpretation provided.
3. Focused on a specific zoonotic disease or general zoonosis prevention and control in a defined time or a period and setting.
4. No limits in types of study but methods should be provided, such as primary research, case study, or literature review.
5. Full text available in English.

### Exclusion criteria

To exclude articles without contents or analysis on social and societal aspects, exclusion criteria were defined as follows:

1. Studies without focusing on analysing the OH implementation from an insight relate to social science would be excluded.
2. Studies would be excluded if without presenting evidence or examples on the OH implementation in zoonotic diseases prevention and control, nor without finding on its social or societal influence.

### Analytical approach

According to a discourse analysis proposed by Galaz et al. [21], three narrative themes were used to describe OH from the lens of social science: integration (scientific and political influence on a broad scale) [22], economics (financial influence, including budgets, cost-effectiveness, and resource allocation), and local context (social impacts, including community-level social, behavioral, and structural dynamics). These themes were used to analyze three different aspects of articles included in the review. Qualitative data analysis software NVivo plus 11.3.0.773 for Windows (QSR International Pty Ltd., Melbourne, Australia) were used to manage qualitative materials in reviewed articles.

## Result

### Overview of studies included

**Fig. 1:**
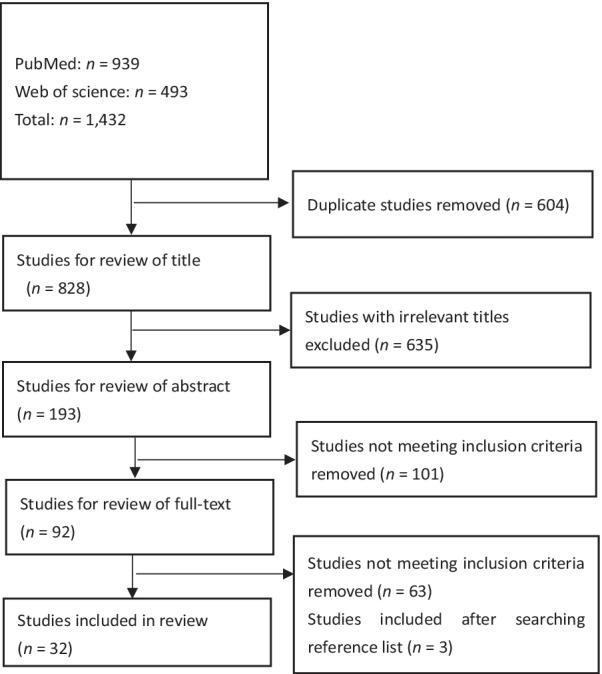
Literature search and review process

A total of 32 studies met the eligibility criteria and were included in the scoping review, in which three studies were included after searching reference list of included studies (Fig. 1). Most articles (24/32) were published between 2014 and 2021. The most common methods were qualitative or semi-qualitative, including case studies (11/32), semi-structured or in-depth interviews and questionnaires (10/32), general literature reviews (4/32), and ethnographic studies (2/32). Some studies used mixed methods to research social determinants, such as combining Delphi technique with qualitative interviews (2/32). Quantitative methods were also used to research zoonotic diseases (2/32) and assess the economic benefit of OH outcomes (2/32). Twenty studies analyzed OH implementation in the context of zoonosis prevention and control with cases provided. Other studies focused on a particular zoonotic disease, such as rabies and brucellosis. Nineteen studies were conducted in low- and middle-income countries (LMICs), including 12 in Africa and eight in Asia (one study conducted in both continents). Six studies were conducted in high-income countries [Australia (*n* = 4), Singapore (*n* = 2), and Switzerland (*n* = 1)]. Some studies did not mention study settings but evaluated cases in Africa or LMICs. It can be seen from included studies that various impacts of OH implementation were demonstrated from the perspective of ‘integration’, ‘economic impacts’, and ‘social determinants of health’ (Tables 1 and 2).

**Table 1:** Impacts of OH implementation focus

Zoonotic diseases focus		Social-related impacts of OH approach on zoonosis prevention and control						
		Integration reflected from OH implementation			Economic impact		Social determinants of health	
		Politics	Disciplines	Communications	Funds & Resources allocation, Cost-effectiveness	Alleviate poverty in a long run	Anthropogenic and ecological factor focused	Fit in cultural and local contexts
General zoonosis		Bardosh et al. [14]; Barnett et al. [37]; Degeling et al. [53]; Dzingirai et al. [41]; Falzon et al. [38]; Johnson et al. [23]; Rweyemamu et al. [54]; Degeling et al. [25]	Bardosh et al. [14]; Barnett et al. [37]; Dzingirai et al. [55]; Johnson et al. [23]	Bardosh et al. [14]; Johnson et al. [23]; Degeling et al. [25]; Rweyemamu et al. [54]	Torgerson [32]; Cunningham et al. [56]; Hasler et al. [30]; Johnson et al. [23]; Welburn et al. [34]; Zinsstag et al. [35]	Torgerson [32]; Dzingirai et al. [55]; Hasler et al. [30]; Welburn et al. [34]; Zinsstag et al. [35]	Saylors et al. [36]; Bardosh et al. [14]; Barnett et al. [37]; Binot et al. [42]; Cunningham et al. [56]; Dzingirai et al. [41]; Johnson et al. [23]; Woldehanna and Zimicki [57]; Lysaght et al. [40]; Zinsstag et al. [35]; Falzon et al. [38]	Bardosh et al. [14]; Barnett et al. [37]; Cunningham et al. [56]; Woldehanna and Zimicki [57]; Yasobant et al. [58]
Specific zoonotic diseases	Brucellosis	Buttigieg et al. [59]; Hermesh et al. [26]	Hermesh et al. [26]	Kakkar et al. [29]	Kakkar et al. [29]	Kakkar et al. [29]	Buttigieg et al. [59]	Kakkar et al. [29]; Hermesh et al. [26]
	*Echinococcus granulosus*				Kakkar et al. [29]	Kakkar et al. [29]		Kakkar et al. [29]
	HPAI A(H5N1)	Chien [44]	Okello et al. [27]	Okello et al. [27]; Chien [44]	Okello et al. [27]		Chien [44]	Chien [44]
	Rabies	Bardosh et al. [60]	Hasler et al. [28]		Hasler et al. [28];	Hasler et al. [28];	Hasler et al. [28]; Bardosh et al. [60]	Okello et al. [27]
	*Campylobacter*		Babo Martins et al. [33]		Babo Martins et al. [33]	Babo Martins et al. [33]		
	*Taenia* *solium*		Bardosh et al. [39]				Bardosh et al. [39]	Bardosh et al. [39]
	Anthrax (*Bacillus anthracis*)		Coffin et al. [61]		Coffin et al. [61]		Coffin et al. [61]	
	MERS-CoV	Farag et al. [24]	Farag et al. [24]	Farag et al. [24]	Farag et al. [24]			
	Sleeping sickness (Human African Trypanosomiasis)	Okello et al. [27]			Bardosh [62]; Okello et al. [27]		Bardosh [62]	Bardosh [62]
	Hendra virus	Landford and Nunn [63]	Landford and Nunn [63]		Landford and Nunn [63]		Landford and Nunn[63]	Landford and Nunn [63]

**Table 2:** Literature's main findings regarding social-related impacts of OH approach on zoonosis prevention and control

Main findings		Literature support
Integration reflected	Political will from decision-makers with various backgrounds and interests and their political involvement are necessary factors in OH implementation	Johnson et al. [23]; Farag et al. [24]; Degeling et al. [25]; Hermesh et al. [26]; Degeling et al. [53]; Rweyemamu et al. [54]; Landford and Nunn [63], Landford and Nunn [63]
	The engagement and participation of multiple disciplines can better support OH implementation	Hermesh et al. [26]; Okello et al. [27]; Hasler et al. [28]; Babo Martins et al. [33]; Barnett et al. [37]; Dzingirai et al. [55]; Coffin et al. [61]; Landford and Nunn [63]
	The integration can strengthen the communication between different actors involving in OH implementation, and lead to better understandings and outcomes of related works	Bardosh et al. [14]; Farag et al. [24]; Degeling et al. [25]; Kakkar et al. [29]; Chien [44]; Rweyemamu et al. [54]
Economic impacts	OH approach can directly help with distribution of funds, allocation of resources, and consideration of cost-effectiveness within a health program implementation	Farag et al. [24]; Hasler et al. [28]; Kakkar et al. [29]; Hasler et al. [30]; Torgerson et al. [32]; Landford and Nunn [63]
	OH approach can be further expected to alleviate burden of diseases and poverty in the future, even though more direct financial costs might need to be input at the initial stage	Hasler et al. [28]; Kakkar et al. [29]; Torgerson et al. [32]; Babo Martins et al. [33]; Welburn et al. [34]; Zinsstag et al. [35]
Social determinants of health considered by OH	OH approach considers anthropogenic, ecological factors and other social determinants of health related to zoonosis transmissions in-depth and provide more holistic ways to assess and address related risks	Bardosh et al. [14]; Hermesh et al. [26]; Saylors et al. [36]; Barnett et al. [37]; Falzon et al. [38]; Bardosh et al. [39]; Lysaght et al. [40]; Binot et al. [42]; Buttigieg et al. [59]; Bardosh et al. [60]; Coffin et al. [61]
	OH approach can fit into different regions and environments by understanding cultural and local contexts, as well as traditional or minority customs	Bardosh et al. [14]; Kakkar et al. [29]; Barnett et al. [37]; Bardosh et al. [39]; Chien [44]; Cunningham et al. [56]; Woldehanna and Zimicki [57]; Yasobant et al. [58]; Bardosh et al. [62]

### Integration reflected from OH implementation on zoonosis prevention and control

#### Political integration

Various literatures mentioned the interaction of several factors that were brought together for OH implementation. “Political will” were seen to hold strong influence over different actors and their conversations in public health and policy [23–25]. The political will to develop health programs and strategies is extremely important. As demonstrated in the Medicaid project in the United States [25], the mobilization of projects and resources transcends the health sector as the decisions come from leaders and politicians. Political involvement is absolutely necessary to overcome operational barriers, which is essential to involve relevant politicians and ministers as they make crucial decision about the scalability of health promotion projects [23]. Farag et al. [24] showed how in the Middle East political will played a key role in influencing OH implementation. It specifically outlined that poor leadership and the absence of committees involved in infection prevention and control helped spread Middle East respiratory syndrome coronavirus (MERS-CoV), limiting the implementation of OH.

Another key factor was the engagement of stakeholders in OH projects. Various actors cooperate to support the health sector and develop health programs. Hermesh et al. [26] pointed out that these actors, such as decision- and policy-makers who can drive the political will, must take their responsibility with more active engagement instead of a passive presence. For instance, in the brucellosis intervention campaign, these actors jointly elaborated effective long-term strategies by considering different barriers and allowed the implementation of sustainable and ethical practices in disease prevention.

#### Interdisciplinary integration

It is imperative that stakeholders and donors must understand their roles and responsibilities in OH implementation. It is the engagement of global donors that can give impetus to the research on global health issues and pushed it into higher levels [27]. It increased the interest in disease prevention and control, thus supported small scale local OH projects and studies to address health crises with the engagement and participation of multiple disciplines [27]. It was noticed that economists and social scientists contributed significantly to designing programs to decrease the risk of HPAI A(H5N1) infection in Nigeria, Tanzania, and Uganda [27]. For example, economists can help understand the disease burden on the national and the individual level, as well as help elicit how poverty and unemployment act as catalysts to the spread of diseases, and social scientists can help analyze how human activities, including their economic conditions, cultural practices and social trends, contributed to the spread of diseases [26]. Moreover, in a case of rabies control in Colombo City, Sri Lanka, OH framework integrating methods and data from multiple disciplines provided decision-makers with relevant information[28]. Thus, it was important to onboard people from various academic fields to build up a health program.

#### Cross-sectoral integration

Communication is a key problem which was addressed by the integration of individuals from varying fields [29]. A study conducted by Bardosh et al. [14] mentioned how medical researchers failed to see the social contexts of the study when making suggestions. The suggestions made by the researchers was impractical as they failed to account for regional social organization of people, power dynamics, socio-cultural norms, and etc. Degeling et al. [25] also identified the broader reach of the social sciences which go beyond just suggestions of technologies and hygiene practices and were more policy focused. It was pointed out that with the integration of the social sciences the OH approach allows for better communication among different sectors and a broader understanding of causality. Thus, the social determinants of health are integral to health promotion and OH implementation. In addition, communication between researchers and sectors seems relatively essential in managing health issues, and OH is greatly helpful in strengthening the communication by collaborating sectors, academics and individuals.

### Economic impacts of OH implementation on zoonosis prevention and control

#### Financial resource allocation

An essential part of OH implementation is its funding. Farag et al. [24] found that the financial resource allocation played a pivotal role in the MERS-CoV programs, as the cost of OH programs had been underestimated, leading to poor management. Underfunding was attributed to miscalculations and the disproportionate allocation of funds among different sectors, decreasing the effectiveness of control programs. This proves that not only it is essential to understand the cost of technology and pharma involved but also to understand the costs of management, organization and operation. This was further expanded in a study [30] that the stakeholders must be willing to invest resources in the OH program and its activities. These activities may not be entirely predictable but if succinct they will have a potential to provide large benefits to the prevention of zoonotic disease. Zoonotic disease also impacts markets through price mechanisms. It was noticed that social planners of OH programs should consider the social cost of a disease to prevent market failure which may further leads to the unavailability of resources [30]. For instance, in the livestock industry, a radical decrease in the availability of meat products would lead to market failures, recession, and poverty, thus increasing disease risk [30, 31].

#### Long-term economic impacts

The included studies provided some long-term economic impacts of the OH implementation and its complexity, including cost–benefit ratio and other monetary and non-monetary outcomes. There are snowball effects to the economics and society that disease and poverty as multidimensional social phenomenon are enclosed in a positive feedback loop that the worse of one may results in the exacerbation of the other [29]. For example, on the one hand, livestock industries provided value to society in the form of food, agriculture, employment and producing revenue, on the other hand, the loss of livestock due to zoonotic diseases can pose adverse impact to human societies in these areas [32]. Though current available evidence may not be enough to demonstrate the impact of OH in alleviating poverty, the correlation between OH implementation and poverty alleviation is becoming more obvious, because OH may be potential to reduce the economic burden of disease and generate more efficient systems.

OH programs have direct and indirect costs in human societies [27, 30]. The costs of death, sickness and injury and the costs of treating the disease were valued as direct costs of a health intervention, while indirect costs are more difficult to estimate, including the loss of wages to workers who are sick and the reduced productivity of workers who may have sub-clinical effects of disease [32]. Similarly, the indirect impacts of OH implementation were described as societal benefits including improved governance, increased social acceptance of interventions and social equity [29], and direct impacts were economic benefits and a reduction in disease burden, such as fluctuations in costs and disability-adjusted life years (DALYs) [32, 33]. According to several case studies on neglected zoonotic diseases, the costs of a OH program can be outweighed after taking these monetary and non-monetary benefits into account [34].

In determining the potential effectiveness of OH programs, the indirect costs and impacts may be more important than the direct ones. It is indicated that economic benefits in the form of a reduced disease burden can be evidently seen when there is a long-term inter-sectoral approach to be implemented [33]. Nonetheless, tangible benefits of OH might be vague at the initial stage as OH implementation tend to cost more financial resources at first and show positive effects after a long time [35]. In the assessment on *Campylobacter* surveillance in Switzerland [33], although the direct effect and measurable benefit were reported being intangible in the initial 5 years of the program, its positive effects were estimated to increase with time [33]. Similar results were found in rabies control in Colombo City, where for the four-year time period the OH interventions cost nearly 1 million US dollars more than their previous program. But apart from reducing dog rabies cases, OH also achieved in reducing people’s distress due to dog bites and animal suffering, and led to positive changes in society [28]. Overall, the value of OH’s potential achievements can exceed the monetary cost of the program and present its overall worth, thus it is better for decision-makers to implement OH programs that can positively affect markets and societies [29].

### Social determinants of health considered by OH approach

#### Improving health through social perspectives

It is fundamental to adopt a multidisciplinary approach to explore the social dimensions and human behaviors associated with disease transmission and understand the conditions and circumstances in which zoonotic diseases emerge and spread [36]. Thus, OH programs can be better understood from a social perspective [37]. This perspective can help promote OH programs to the public and improve them at the level of governance, which can further influence power and politics. OH implementation is also expected to achieve better local or regional understandings and capacities from social insights [14, 38].

From the lens of social determinants of health, OH proved that anthropogenic factors contribute to the spread of zoonotic pathogens to humans, such as high-risk lifestyles, intensive livestock production, exhaustive agricultural practices, urbanization, globalization, and pollution [36]. These factors were also found to be interrelated with social classes and socioeconomic status in human society [39]. Thus making zoonotic disease transmission not only related to its pathophysiology but also to certain social determinants, such as social norms, economic imperatives and human values, which shows the pattern that humans interact with animals [40]. For example, the risk of infection with Rift Valley fever in Kenya was found to be strongly linked to the socioeconomic status of affected and at-risk communities, as people of lower socioeconomic status were more likely to be exposed to environments full of mosquitoes [41]. To minimize the health gap between communities with different socioeconomic status, it is important to consider the difference in their social needs when conducting health interventions. It is also acknowledged that zoonotic diseases transmission is affected by ecological and social dynamics, thus analyzing epidemiology patterns with ecological and social factors is needed [26, 42]. For instance, climate change in tropical areas is associated with the emergence and spread of zoonotic pathogens. Additionally, the ecotourism can increase the risk of zoonotic disease transmission and spread over long distances [42].

#### Implementing OH under local contexts

Understanding local contexts and behavioral patterns that affect disease transmission can help improve the response efforts and design culturally-acceptable interventions. For instance, in Lao Peoples' Democratic Republic (Lao PDR), social determinants such as poor latrines coverage, limited access to clean water, and consumption of raw pork meat can increase the risk of *Taenia solium* infection [39]. In addition, the trust between health workers and local leaders were found to be essential in better performing the intervention [39]. For example, because of anthropologists’ insights and research on local societal hierarchy and social practices, a better cooperation between local communities and international health workers were achieved, which increased the effectiveness of controlling Ebola outbreaks in Africa[43]. These cases demonstrated the effectiveness of OH programs depends on understanding local systems [37]. The social and cultural factors complicate disease transmission and the implementation of health interventions [44], thus it is necessary to include social and ethnographic study into OH implementation.

Moreover, LMICs were found to be more susceptible to infectious disease, economic vulnerability and food security, which may be due to limited financial resources and governance capacity [29]. The health system in many LMICs is either out of pocket or subsidized thus marginalizing the communities of lower socioeconomic status. Therefore, in LMICs, OH is adopted to provide an in-depth understanding of the economic feasibility of projects and increase the availability of local resources [14]. The OH approach takes into account how complex the issue and program planning can be. It also holds responsibility for cost saving and using the limited resources effectively.

## Discussion

### OH as a discourse model

As a concept, ‘One Health’, expanding from ‘One Medicine’, has been obtaining various features and attributes from different intellectuals and academic fields. OH used to focus on zoonosis that are infectious diseases, then gradually focused on chemical- or poison-related illnesses in animals and their relationship to the detection and prevention of human illness. This have made OH become a hot topic and buzzword, producing its value by being the term with significance in both scientific and societal worlds [9]. It was also described as a generalized and flexible term that captures the will to address the complexities and interrelations that exist between human, animal and ecological health. This kind of discourse influence can be seen from ‘One Toxicology’ [45], which adopted the ‘One Health’ language, indicating shared sources of food and water for humans and animals are a common route of toxin exposure. Another emerging term from ‘One Health’ can be seen in ‘One Welfare’, which shared OH ideas and applied them into welfare issues, referring to animal and human welfare, as well as societal mental health and environmental conservation [46]. It is also considered working together with OH approach to better serve the society [46]. The influence of OH as a crucial term in this topic is shown to have the ability to improve global public health awareness and response [45].

### Underlying barriers for OH implementation

For national governments, managing zoonotic diseases through OH depends on implementing comprehensive public health strategies by considering various social determinants of health [22]. However, the policy challenges of integrating animal health and public health priorities in the context of trade and development were reported to remain relatively unexamined [47]. It was observed that lacking evidence of OH implementation resulted a failure to measure the impact of OH programs, which limited the promotion of OH and adoption of sustainable policy [29]. In addition, the level of cooperation between scientists from different disciplines depends on the complexity of health issues [42]. There was also lack of a congruency of thought and agreeability of action within the individuals if any conflict of interest occurred. It indicated the importance to develop a common goal and systems thinking in order to better implement the OH.

As aforementioned, a large problem in the OH implementation is the lack of political will. As the foundation of political involvement, the absence of political will may limit the organization, implementation, and scalability of OH programs [24]. There also seems to be a lack of socioeconomic evidence to promote the OH implementation [29]. These evidence can provide space for conversations with policymakers and stakeholder. In addition, a lack of evidence in this area reduces the generalizability and real-world applicability of OH, making it remain at the theoretical stage.

### OH’s further development

A severely impacting virus we see nowadays is severe acute respiratory syndrome coronavirus 2 (SARS-CoV-2), and there was a debate around whether COVID-19 should be classified as a new zoonotic disease. A group of researchers argued that the better classification is "emerging infectious disease (EID) of probable animal origin.", because no confirmed case of transmission from animals was reported, and most human COVID-19 infections were through human–human transmission [48]. However, as more and more animals became infected after contact with human who had COVID-19, a lot of researchers considered it to be a zoonotic virus even though animals do not serve as a source or part of COVID-19 infection among people [49]. Despite of the classification of disease, the application OH approach can be significant in making response to the COVID-19 pandemic as a global health issue [50]. OH can support in building integrated surveillance system by collecting data related to infections and risk behaviors in both human and animals, which also requires to improve the coordination across governments and sectors. Moreover, OH considers health equities in solving health issues, recommending policy-makers to make response mechanisms and interventions based on socio-economic contexts, which cover the health needs of individuals and groups in vulnerable conditions [50].

By reviewing studies on OH implementation, it was found that most studies were designed to fit a group of people in a limited geography, and some problems may be difficult to be identified in such pilot studies. Thus, larger studies are necessary to assess the effects of this framework at the national level [51]. A barrier to a successful national OH approach might be that some decision makers’ comprehension of the approach are still vague [40]. This might because there are few clear definitions or policy guidelines for the OH implementation [29], which also becomes a barrier to better achieve interdisciplinary cooperation. However, carrying out large scale studies would require higher financial input. In LMICs where budgets are tight, the implementation of OH might be limited without enough academic and technological resources. To achieve the goal of global health, it is important for international organizations and developed countries to support and cooperate with LMICs in OH implementation [29, 38].

To summarize, there seems to be a discrepancy between theory and practice of OH. Nevertheless, the OH framework improves human and environmental health by adopting a systems approach. The cooperation between sectors and disciplines allows for a better policy-making process and a more holistic view on zoonosis prevention and control. Moreover, the One Health High Level Expert Panel (OHHLEP) composed of the WHO, FAO, OIE, and the United Nations Environment Programme (UNEP) recently defined OH as ‘an integrated, unifying approach that aims to sustainably balance and optimize the health of people, animals and ecosystems.’, which recognizes the importance and responsibilities of the health of humans, domestic and wild animals, plants, and the wider environment [52]. It is expected to integrate other sectors, disciplines, and communities to promote well-being and sustainable development and reduce health and ecological risk [52].

## Limitation

One of the limitation of this study is that it mostly included qualitative studies and materials rather than quantitative, which may potentially lead to analytical bias. Another limitation was it only reviewed literature in English, which may miss some relevant insights of this topic in non-English languages using countries.

## Conclusions

The OH approach adopts interdisciplinary practices to improve zoonosis prevention and control by reducing risks at the human-animal-environment interface and addressing social determinants of health. Integrating social sciences in OH implementation can better addressing concerns of employment, economics, culture, real life experiences and politics. The OH approach penetrates diseases causality in depth by impacting the social aspects at the local, national and global level. The OH implementation also needs to overcome political barriers. Although evidence on the effectiveness of OH for zoonosis prevention and control is still limited, the available evidence in pilot programs and cases showed promising results. Global health issues are becoming increasingly complex, it is expected that comprehensive OH approach will be adopted worldwide to mitigate these issues, with significant benefits to society.

## Data Availability

All data generated or analyzed during this study are included in this published article.

## Abbreviations

OH: One Health
WHO: World Health Organization
OIE: World Organization for Animal Health
FAO: Food and Agricultural Organization of the United Nations
LMICs: Low- and middle-income countries
MERS-CoV: Middle East Respiratory Syndrome Coronavirus
HPAI A(H5N1): Highly pathogenic Asian avian influenza A (H5N1) virus
DALYs: Disability-adjusted life years
Lao PDR: Lao Peoples' Democratic Republic
SARS-CoV-2, COVID-19: Severe acute respiratory syndrome coronavirus 2
EID: Emerging infectious disease
OHHLEP: One health high level expert panel
UNEP: United Nations Environment Programme
